# Autophosphorylation Mechanism of the Ser/Thr Kinase Stk1 From *Staphylococcus aureus*

**DOI:** 10.3389/fmicb.2018.00758

**Published:** 2018-04-20

**Authors:** Weihao Zheng, Xiaodan Cai, Shuiming Li, Zigang Li

**Affiliations:** ^1^School of Chemical Biology and Biotechnology, Peking University Shenzhen Graduate School, Shenzhen, China; ^2^College of Life Sciences and Oceanography, Shenzhen University, Shenzhen, China

**Keywords:** Ser/Thr kinase, Stk1, autophosphorylation, activation loop, phosphoproteomic, *Staphylococcus aureus*

## Abstract

The eukaryotic-like Ser/Thr kinase Stk1 is crucial for virulence, cell wall biosynthesis, and drug susceptibility in methicillin-resistant *Staphylococcus aureus* (*S. aureus*) (MRSA). Importantly, MRSA lacking Stk1 become sensitive to β-lactam antibiotics, implying that Stk1 could be an alternative target for combination therapy. However, the autophosphorylation mechanism of Stk1 remains elusive. Using a phosphoproteomic study, we identified six *in vivo* phosphorylated activation loop residues (Ser159, Thr161, Ser162, Thr164, Thr166, and Thr172) of Stk1, which are also phosphorylated *in vitro*. We further showed that *cis* autophosphorylation of Thr172 in the GT/S motif is essential for self-activation and kinase activity of Stk1 kinase domain (Stk1-KD), whereas the *trans* autophosphorylation of other activation loop serines/threonines are required for the optimal kinase activity of Stk1-KD. Moreover, substitution of the activation loop serines/threonines impaired *in vivo* autophosphorylation activity of kinase variants, while T172A and T172D variants were unable to autophosphorylate in the cellular content, underlining the essential role of Thr172 for Stk1 activity *in vivo*. This study provides insights into molecular basis for regulation of Stk1 activity from *S. aureus*.

## Introduction

*Staphylococcus aureus* (*S. aureus*) is one of the major human pathogens that greatly impacts individuals and causes a variety of illnesses ranging from minor skin infections to life-threatening diseases, such as endocarditis, pneumonia, septicemia, and toxic shock syndrome (Lowy, 1998). This pathogen persistently colonizes about 20% of the human population and can infect almost every tissue in the human host (Kluytmans et al., 1997). Antibiotic treatment is often ineffective owing to the generation of multiple drug-resistant strains, including methicillin-resistant *S. aureus* strains (MRSA) and vancomycin-resistant *S. aureus* strains (Gardete and Tomasz, 2014; Peacock and Paterson, 2015). New strategies for combating *S. aureus* infection are urgently needed. Thus, better understandings of molecular basis of important components for pathogenesis and virulence regulation are of crucial importance.

To survive, bacteria have evolved multiple signal transduction systems to sense the environmental stimuli including nutrient concentrations and oxygen tension, eliciting appropriate activation or inactivation of response regulators (Rakette et al., 2012; Wright and Ulijasz, 2014). This is generally achieved through reversible protein phosphorylation mediated by protein kinases/phosphatase pairs, including well-known bacterial signaling cascades of two-component systems (TCSs). TCSs are constituted by His/Asp-based phosphorelay systems that consist of sensor histidine kinases and cognate DNA-binding response regulators (Zschiedrich et al., 2016). Recently, eukaryotic-like Ser/Thr kinases/phosphatases (eSTKs/eSTPs) were found to be another conserved and critical signal transduction system in bacteria. eSTKs/eSTPs regulate many aspects of bacterial physiology including virulence, cell division, antibiotic resistance, secondary metabolism, and host–pathogen interactions (Ohlsen and Donat, 2010; Burnside and Rajagopal, 2011; Pereira et al., 2011; Wright and Ulijasz, 2014).

**FIGURE 1:**
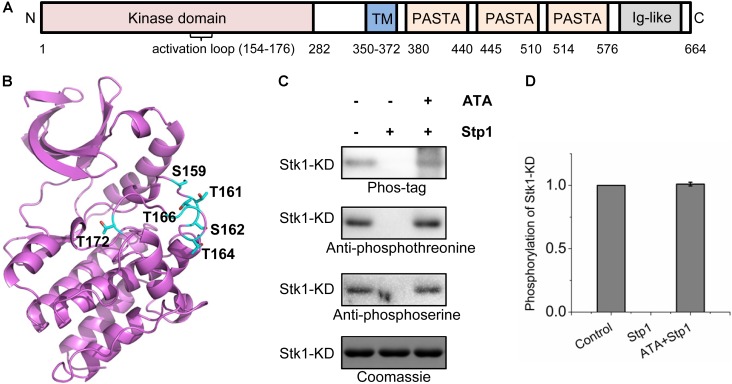
Identification of *in vivo* phosphorylated residues within the activation loop of Stk1. **(A)** Topology of *Staphylococcus aureus* Stk1. TM, transmembrane domain; PASTA, penicillin-binding protein and serine/threonine kinase-associated domains; Ig-like, immunoglobulin-like domain. **(B)** Location of the autophosphorylated residues (colored cyan) in the activation loop of Stk1. The Stk1-KD model was built by using Swiss-model, with Mtb-PknA (PDB code: 4OW8) as a template. **(C)** Role of Stp1 in the dephosphorylation of autophosphorylated residues of Stk1-KD. 0.5 μg Stk1-KD proteins were incubated with ATP at 37°C for 30 min, followed by addition of Stp1 (0.2 μM), or ATA (400 μM) and Stp1 (0.2 μM) for an additional 1-h incubation. Reaction mixture without Stp1 and ATA served as control. All samples were analyzed by western blotting. Protein phosphorylation was detected using Phos-tag-bound Streptavidin-conjugated HRP (Phos-tag), anti-phosphothreonine antibody, or anti-phosphoserine antibody. To ensure loading quality, similar amounts of Stk1-KD were subjected to electrophoresis on a 12% SDS-PAGE gel and stained by Coomassie R250. **(D)** Relative quantification of autophosphorylation activity of Stk1-KD. Data are represented as the mean ± SEM, *n* = 3 independent experiments.

*Staphylococcus aureus* possesses a sole pair of eSTK/eSTP, designated Stk1/Stp1, which play key roles in cell wall metabolism, virulence, and drug resistance (Beltramini et al., 2009; Debarbouille et al., 2009; Donat et al., 2009; Burnside et al., 2010; Liebeke et al., 2010; Tamber et al., 2010; Zheng et al., 2016, 2017; Cai et al., 2017). Based on the presence of Arg which precedes a conserved catalytic Asp, STKs can be classified into RD and non-RD kinases (Johnson et al., 1996). Stk1 is a RD-family kinase, and contains an N-terminal, intracellular kinase domain, a hydrophobic transmembrane domain, and three extracellular PASTA (for penicillin-binding protein and Ser/Thr kinase-associated) domains and an Ig-like domain at C-termini (**Figure 1A**). PASTA domains are composed of ∼65 amino acids and are thought to be a sensor motif to bind beta-lactam compounds as well as cell wall fragments (e.g., peptidoglycans) (Yeats et al., 2002; Hardt et al., 2017). Intriguingly, deletion of *stk1* renders MRSA to become susceptible to β-lactam antibiotics (Beltramini et al., 2009; Tamber et al., 2010), indicating that Stk1 could be a potential target for combination therapy. So far, biochemical and genetic studies have revealed physical roles of Stk1 (Beltramini et al., 2009; Debarbouille et al., 2009; Donat et al., 2009; Burnside et al., 2010; Tamber et al., 2010). However, regulation of its activity is unknown.

In this study, we focus on investigating the autophosphorylation mechanism of Stk1. Using mass spectrometry studies, we found six residues (Ser159, Thr161, Ser162, Thr164, Thr166, and Thr172) located at the activation loop are phosphorylated both *in vivo* and *in vitro*. Mutagenesis and biochemical studies revealed the involvement of these residues in autophosphorylation and kinase activities. We demonstrated that Thr172 is crucial for activity of Stk1 both *in vitro* and *in vivo*. Our findings comprise a step toward understanding the activation of RD-family kinases of eSTKs.

## Materials and Methods

### Cloning and Mutagenesis

The primers used in current study are listed in Supplementary Table S1. With primers Stk1KD-NdeI-F and Stk1KD-XhoI-R, kinase domain of *stk1* (Stk1-KD, residues 1–296) was amplified from a previous plasmid pET22b-*stk1* carrying full length of *stk1* (Zheng et al., 2015). The purified PCR products were digested with NdeI and XhoI, then ligated into NdeI/XhoI sites of pET28a, resulting in pET28a-*stk1-KD*. Conventional site-directed mutagenesis was conducted to construct all Stk1-KD variants including 5M (S159A/T161A/S162A/T164A/T166A) with desired point mutation(s). All of the constructs were sequenced to rule out unwanted mutations.

### Protein Expression and Purification

The expression and purification of kinases and Stp1 have been described previously (Zheng et al., 2015, 2016). Briefly, BL21 (DE3) pLysS cells carrying pET28a constructs were cultured in one liter of LB medium at 37°C. After OD_600_ reached 0.6–0.8, the cells were induced with 0.5 mM isopropyl-β-D-thiogalactopyranoside (IPTG) at 16°C for overnight. Bacteria were collected by centrifugation and re-suspended in 30 mL of buffer A [50 mM Tris-HCl, pH 8.0, 500 mM NaCl, 50 mM imidazole, and 5% (wt/vol) glycerol] with protease inhibitor phenylmethanesulfonyl fluoride (PMSF). After loaded to a HisTrap HP column, the column was washed with appropriate volume of 0 and 5% (v/v) buffer B [50 mM Tris-HCl pH 8.0, 500 mM NaCl, 500 mM imidazole, and 5% (wt/vol) glycerol]. Then the protein was eluted with a 50% (v/v) buffer B. The fractions were collected, concentrated, and further purified by a HiTrap desalting column with desalting buffer I (20 mM Tris-HCl, pH 7.5, 150 mM NaCl).

To prepare His-tag-free T172A (fT172A), the His-tag of T172A was removed by thrombin (Novagen) cleavage for 20 h at room temperature (RT). The protein solution was passed through a HisTrap HP column to remove the His-tag, and was further purified by a Superdex 200 10/300 GL column with desalting buffer I. All the purified proteins were >90% pure as estimated by the 12% (wt/vol) SDS-PAGE gel (Supplementary Figure S5). All of the purified proteins were aliquoted and stored at -80°C until used.

### Protein Kinase Assays

To investigate the effect of single-point mutation on autophosphorylation activity and kinase activity of Stk1, 2 μg kinase was first incubated in 20 μL of buffer P (50 mM Tris-HCl, pH 7.5, 2 mM MnCl_2_ and 0.2 mM ATP) at 37°C for 30 min, followed by the addition of 8 μg MBP (Sigma) and another 30-min incubation at 37°C. To examine the trans-autophosphorylation activity of WT, 5M and T172D toward fT172A, 0.2 μg WT or 5M or T172D was incubated with 1.5 μg fT172A at RT for 30 min, then 8 μg MBP was added subsequently for additional 15-min incubation at 37°C. All reactions were quenched with the addition of 4× SDS-PAGE loading buffer. Samples were separated by electrophoresis on 12% SDS-PAGE gel and transferred to a PVDF membrane. After equilibrating with TBST buffer for 1–2 h, the membrane was probed with Phos-tag-bound Streptavidin-conjugated HRP [prepared from Phos-tag Biotin (Wako)] at RT for 30 min. After incubation, the membrane was washed twice for 5 min each time in TBST buffer and detected by chemiluminescent reaction with ChemiDoc XRS System (Bio-Rad).

### Phosphorylation of Stk1-KD Variants in *E. coli* Cells

Phosphorylation levels of Stk1-KD variants were determined by western blotting analysis. BL21 (DE3) pLysS cells harboring pET28a-*stk1-KD*, pET28a-*stk1-KD* S159A, pET28a-*stk1-KD* T161A, pET28a-*stk1-KD* S162A, pET28a-*stk1-KD* T164A, pET28a-*stk1-KD* T166A, pET28a-*stk1-KD* T172A, pET28a-*stk1-KD* T172D, pET28a-*stk1-KD* 5M, or pET28a vector alone were overnight cultured and 100-fold diluted into 10 mL fresh LB medium containing 50 μg/mL Kanamycin and 34 μg/mL Chloramphenicol. After OD_600_ reached 0.6–0.8, the cells were induced with 1 mM IPTG at 37°C for 5 h. Cells were harvested by centrifugation, washed with PBS and resuspended in PBS contaning 20 mM EDTA, and 1 mM PMSF, and were lysed at 4°C by sonication. The supernatant was passed through the 0.2 μm filter before being collected. 15 μg of total protein lysates were separated by electrophoresis on 12% SDS-PAGE gel and analyzed by western blotting using Phos-tag-bound Streptavidin-conjugated HRP or mouse anti-His antibody (1:5000, MBL).

### Liquid Chromatography–Mass Spectrometry/Mass Spectrometry (LC–MS/MS) Analysis

Liquid chromatography–mass spectrometry/mass spectrometry (LC–MS/MS) analysis was performed as previously described with modifications (Cai et al., 2018). After *in vitro* phosphorylation with non-radioactive ATP, the mixture containing purified Stk1-KD was subjected to electrophoresis on a 12% SDS-PAGE gel, which was stained by Coomassie R250. The gel band corresponding to Stk1-KD was cut into pieces, subjected to reduction with 10 mM DTT at 56°C for 50 min, and subsequent alkylation with 55 mM iodoacetamide (IAA) for 30 min at RT in dark. Then in-gel digestion was performed with trypsin [Promega, enzyme: protein = 1:50 (wt/wt)] at 37°C for 18 h in 25 mM ammonium bicarbonate buffer. The lyophilized tryptic digested samples were re-dissolved in 2% acetonitrile, 0.1% formic acid, and loaded on ChromXP C18 (3 μm, 120 Å) nanoLC trap column. The online trapping, desalting procedure was carried out at a flow rate of 2 μL/min for 10 min with 100% solvent A (Solvent A: water/acetonitrile/formic acid = 98/2/0.1% solvent B: 2/98/0.1%). Then, an 60-min gradient elution ranging from 5 to 35% acetonitrile (0.1% formic acid) was used on an analytical column (75 μm × 15 cm C18- 3 μm 120 Å, ChromXP Eksigent). LC–MS/MS analysis was performed with a Triple TOF 5600 System (AB SCIEX, Concord, ON, Canada) fitted with a Nanospray III source (AB SCIEX, Concord, ON, Canada). Data was acquired using an ion spray voltage of 2.5 kV, curtain gas of 30 PSI, nebulizer gas of 5 PSI, and an interface heater temperature of 150°C. The MS was operated with TOF-MS scans. For IDA, survey scans were acquired in 250 ms and as many as 25 product ion scans (90 ms) were collected if exceeding a threshold of 150 counts per second (counts/s) and with a +2 to +4 charge-state. A Rolling collision energy setting was applied to all precursor ions for collision-induced dissociation. Dynamic exclusion was set for ½ of peak width (∼12 s). For data analysis, the.wiff file was processed by ProteinPilot 5.0. Searches were performed against the local database including the protein sequences for Stk1-KD and decoys. The search effort was set to ‘Thorough ID’ and the False Discovery Rate Analysis was involved with the default setting for ‘Detected Protein Threshold [Unused ProtScore (Conf)]’ at 0.05. Only phosphopeptides with 99% confidence were accepted and further checked manually.

### Phosphoproteomic Analysis

*Staphylococcus aureus* Newman cells were grown at 37°C overnight in tryptic soy broth (TSB) containing 5 μg/mL nalidixic acid. The overnight culture was diluted 100-fold in fresh 100 mL of TSB, and incubated at 37°C, 250 rpm for 5 h. 10 μM Mn^2+^ was added at 1 h before cell harvest. Bacteria were collected by centrifugation, washed quickly once with 20 mL of ice-cold Tris-bufer (pH 8.0) and re-suspended in 5 mL of ice-cold lysis buffer [20 mM Tris-HCl, pH 8.0, 8 M urea, 2 mM a Na_3_VO_4_, 2 mM sodium pyrophosphate and 50 μM aurintricarboxylic acid (ATA)]. After the addition of 1.2 g glass beads, the mixture was subjected to vortex for 20 min (1-min vortex, followed by 1-min ice incubation). Then the lysate was centrifuged at 4°C, 14,000 rpm for 30 min. 0.8 mg protein sample was transferred to a 10K Nanosep^®^ Centrifugal Devices (Pall), and centrifuged at 12,000 rpm for 30 min. Reduction was conducted with 100 μL of buffer1 (0.1 M Tris-HCl, pH 8.5, 8 M urea) containing 10 mM DTT for 1 h at 37°C. Subsequent alkylation was performed in buffer2 (buffer1+ 50 mM IAA) for 15 min at RT in dark. After centrifugation at 12,000 rpm for 20 min (remaining volume ∼30 μL), buffer3 (50 mM ammonium bicarbonate) was added for exchanging buffer for three times. The proteolytic digestion was conducted in 200 μL of buffer3 with trypsin [enzyme: protein = 1:50 (wt/wt)] at 37°C for 18 h. After the digested sample was centrifuged at 12,000 rpm for 10 min and washed with 100 μL of buffer3 for twice, all the filtrates were pooled and lyophilized. The trypsin-digested sample was enriched by titanium dioxide beads (GL Sciences). Enriched sample was analyzed by LC–MS/MS as described above. Acquired data were processed by ProteinPilot 5.0 against the local protein database of *S. aureus* Newman (GenBank Accession No.: AP009351.1). Dataset were filtered with the following criteria: for proteins, Unused ProtScore ≥ 2.0, which corresponds to a protein confidence cut-off threshold of 99%; for phosphopeptides, Conf ≥ 99%. Final manual inspection of spectrums was engaged. The mass spectrometry proteomics data have been deposited in the PRIDE Archive^1^ with the project accession number PXD006285 (Username: reviewer52415@ebi.ac.uk; Password: dQQPCCck).

### Circular Dichroism Experiments

Circular dichroism (CD) experiments were conducted as previously described (Zheng et al., 2016). Briefly, the spectra of secondary structure of purified protein were monitored at wavelength range from 195 to 250 nm using a Chirascan spectrometer (Applied Photophysics) at 25°C. Baseline was calibrated by phosphate buffer (10 mM sodium phosphate, pH 7.4) before measurement. Measurements were carried out with 4 μM protein in 0.1-cm path length quartz cuvettes. Three consecutive scans were obtained and averaged for spectra data display.

## Results

### Identification of Phosphorylated Residues on Activation Loop of Stk1 in *S. aureus*

The common regulatory mechanism for activity of Ser/Thr kinases is the phosphorylation of a canonical kinase segment known as the activation loop, which is flanked by the conserved N-terminal DFG and C-terminal APE motifs. Autophosphorylation of activation loop serine, threonine, or tyrosine residues will bring a conformational change that stabilizes the kinase in an active conformation (Nolen et al., 2004). Although this region is highly heterogeneous, it is thought to determine the catalytic state of Ser/Thr kinases and play a key role in the substrate recognition (Nolen et al., 2004; Manuse et al., 2016).

In order to identify the phosphorylation sites on activation loop of Stk1 in *S. aureus*, we conducted a phosphoproteomic study of *S. aureus* Newman strain. The protein extract was first digested with sequencing grade trypsin and enriched by titanium dioxide beads. The isolated phosphopeptides were then subjected to LC–MS/MS analysis. With this approach, we identified 76 peptides with 50 mapped phosphorylation sites belonging to 29 distinct proteins (Supplementary Table S2). 18 phosphorylation sites have been confirmed by other phosphoproteomic studies (Burnside et al., 2010; Basell et al., 2014; Depardieu et al., 2016), indicating the reproducibility of our present study. In the case of Stk1, only one peptide (residues 157–182), with the sequence ALSETSLTQTNHVLGTVQYFSPEQAK, was predicted to have a cluster of six phosphorylation sites (Ser159, Thr161, Ser162, Thr164, Thr166, and Thr172) (**Figure 1B** and Supplementary Figure S1). This segment overlaps with the sequence of kinase activation loop (residues 154–176) in Stk1. We then wondered whether similar phosphorylated residues could be identified after *in vitro* autophosphorylation of kinase domain of Stk1 (Stk1-KD). Purified Stk1-KD was incubated with ATP/Mn^2+^ and subjected to in-gel trypsin digestion. Subsequent LC–MS/MS analysis yielded mono-/di-/triphosphorylated activation loop peptides with same phosphorylation sites as identified *in vivo* (Supplementary Figure S2). These results are also consistent with previous studies where analysis of phosphoamino acids composition revealed that phosphothreonines and, to a lesser extent, phosphoserines were the *in vitro* phosphorylated amino acids of Stk1 (Debarbouille et al., 2009). We further investigated the role of Stp1 in dephosphorylation of Stk1-KD. We found that Stp1 could completely dephosphorylated Stk1-KD during autophosphorylation, while an Stp1 inhibitor ATA inhibited Stp1 dephosphorylating all the phosphorylated residues of Stk1-KD (**Figures 1C,D**). Altogether, these data imply a similar autophosphorylated pattern of activation loop for Stk1 both *in vivo* and *in vitro*. And all the phosphorylated residues of Stk1-KD are the dephosphorylated targets of Stp1.

### Kinase Activities of Stk1 Activation Loop Variants

**FIGURE 2:**
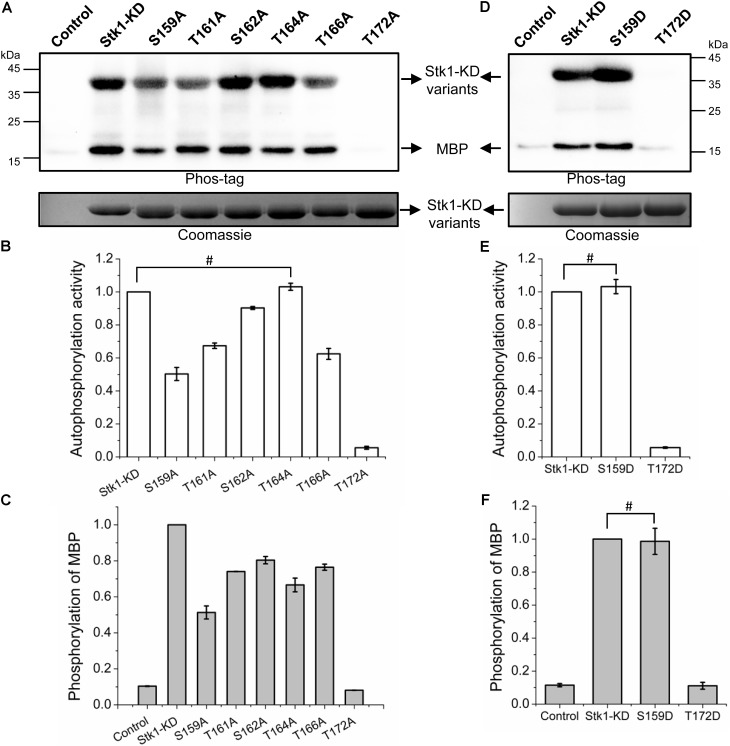
Kinase activities of Stk1 activation loop variants. **(A,D)** Wild type (WT) Stk1-KD or cognate variants (2 μg) were incubated with ATP at 37°C for 30 min, followed by addition of MBP (8 μg) for an additional 30-min incubation. Reaction mixture containing MBP only served as control. All samples were analyzed by western blotting with Phos-tag. To ensure loading quality, similar amounts of protein kinases were subjected to electrophoresis on a 12% SDS-PAGE gel and stained by Coomassie R250. **(B,C,E,F)** Relative quantification of autophosphorylation activity of kinase and MBP phosphorylation. Data are represented as the mean ± SEM, *n* = 3 independent experiments. ^#^*P* > 0.05; two-tailed Student’s *t*-test.

The activation loop serines/threonines are all autophosphorylated, implying that the differential phosphorylation of activation loop may regulates the kinase activity of Stk1. To investigate this possibility, we first explored the contributions of phosphorylation sites of activation loop to kinase activity of Stk1. We generated six Ala substitution variants: S159A, T161A, S162A, T164A, T166A, and T172A. We first examined the autophosphorylation activity of all Stk1-KD variants. As shown in **Figures 2A,B**, S159A, T161A, S162A, and T166A variants exhibited decreased autophosphorylation activity, while T172A variant barely showed any autophosphorylation activity.

To evaluate the substrate phosphorylation activity of Stk1-KD activation loop variants, we used myelin basic protein (MBP) as a surrogate substrate in the *in vitro* kinase assay. Compared to wild type (WT) Stk1-KD, all Stk1-KD variants showed differentially reduced substrate phosphorylation activity, among which T172A variant was not able to phosphorylate MBP (**Figures 2A,C**). We then replaced Ser159 and Thr172 with phosphomimic residue Asp (S159D and T172D) to see if activity of these kinase variants could be rescued. As expected, S159D variant has similar autophosphorylation and substrate phosphorylation activities as the WT kinase, whereas Asp substitution of Thr172 did not restore any activity of the kinase variant despite similar loading of samples in the gel (**Figures 2D–F**). The marginal phosphorylation of T172A and T172D variants may be caused by unknown reasons, which did not activate the autophosphorylation and kinase activities of these variants in the absence of intact Thr172 *in vitro* (**Figure 2**). Collectively, these results suggest that different phosphorylation level of Stk1 activation loop regulates the extent of autophosphorylation and substrate phosphorylation activities of Stk1. In particular, phosphorylation of Thr172 is essential for the functionality of Stk1.

### Stk1-KD and Its Cognate Activation Loop Variants Show Similar Overall Folding

As phosphorylation of activation loop residues will bring conformational changes in Ser/Thr kinase, we next investigated the effect of Ala substitutions of Stk1 activation loop serines/threonines on the overall folding of the proteins. Far-UV (195–250 nm) CD spectroscopy was used to monitor changes in protein secondary structure of Stk1-KD variants. As expected, the CD spectra of WT Stk1-KD was consistent with a mixed α/β fold of structure of Ser/Thr kinases. Ala replacement of Stk1 activation loop serines/threonines caused minor changes in secondary structure content of the kinase variants (Supplementary Figure S3), indicating Ala substitutions do not perturb the overall folding of the enzyme. These data clearly suggest that the reduced activity of Stk1 activation loop variants is not a result of disruption of overall protein conformation.

### Autophosphorylated Mechanism of Stk1

**FIGURE 3:**
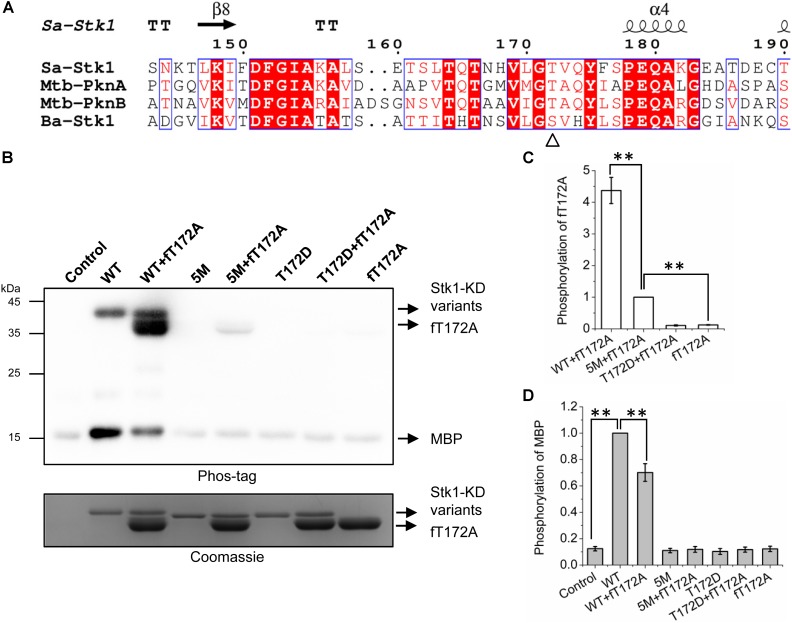
Trans-autophosphorylation of fT172A by WT, 5M, and T172D. **(A)** Multiple sequence alignment of activation loop of Stk1 with homologous eSTKs. Sa, *S. aureus*; Mtb, *M. tuberculosis*; Ba, *B. anthracis*. Secondary structure elements from Stk1 (PDB code: 4EQM) are presented on the top. GT/S motif is indicated by ‘Δ’. **(B)** 0.2 μg WT or 5M or T172D was incubated with 1.5 μg fT172A at RT for 30 min, then 8 μg MBP was added subsequently for an additional 15-min incubation at 37°C. Reaction mixture containing MBP only served as control. All samples were subjected to western blotting analysis. To ensure loading quality, similar amounts of protein kinases were subjected to electrophoresis on a 12% SDS-PAGE gel and stained by Coomassie R250. **(C)** Relative quantification of fT172A phosphorylation. **(D)** Relative quantification of MBP phosphorylation. All data are represented as the mean ± SEM, *n* = 3 independent experiments. ^∗∗^*P* < 0.0001; two-tailed Student’s *t*-test.

In a sequence alignment analysis, it was brought to our attention that Thr172 locates at the conserved GT/S motif presented in the activation loop of homologous bacterial Ser/Thr kinases, whose corresponding serine or threonine are also reported to be phosphorylated (**Figure 3A**) (Mieczkowski et al., 2008; Shakir et al., 2010; Ravala et al., 2015). Indeed, mutagenesis studies have shown that Thr180 of Mtb-PknA from *Mycobacterium tuberculosis* (*M. tuberculosis*) and Ser173 of Ba-Stk1 from *Bacillus anthracis* (*B. anthracis*) are also crucial for kinase activity (Shakir et al., 2010; Ravala et al., 2015). Mtb-PknA structure in unphosphorylated form is the first case with an ordered activation loop in bacterial Ser/Thr kinases (Ravala et al., 2015). To compare the location of Stk1 activation loop serines/threonines, we created a homology model of Stk1-KD (Stk1-KD model) with crystal structure of Mtb-PknA as a template by using Swiss-model. Stk1-KD model shares similar overall folding (RMSD/Cα = 1.385 Å) with the reported crystal structure of Stk1-KD (Stk1-KD structure) (Supplementary Figure S4A) (Rakette et al., 2012). Interestingly, several ordered activation loop residues of Stk1-KD structure adopt similar conformation to that of Stk1-KD model (Supplementary Figure S4A), implying that Stk1-KD model could represent the unphosphorylated form of Stk1-KD. We then modeled ATP and Mn^2+^ into the active site of Stk1-KD model by using Molecular Operating Environment (MOE). After measurement, Thr172 is the nearest residue to the γ-phosphate group of ATP (7.6 Å) compared to other activation loop serines/threonines (14.5–21.5 Å) (Supplementary Figure S4B). These suggest that Thr172 may be first autophosphorylated in *cis* for self-activation of the kinase upon binding with ATP, which may not apply to other activation loop serines/threonines.

To test this hypothesis, we created a new Stk1-KD variant 5M (S159A/T161A/S162A/T164A/T166A) by replacing all the activation loop serines/threonines with Ala except Thr172. His-tag-free T172A (fT172A) was prepared by cleaving the His-tag of T172A (Supplementary Figure S5D). Taking advantage of fT172A variant as the phosphor-recipient, we incubated WT, 5M (Thr172 is intact) and T172D (Ser159, Thr161, Ser162, Thr164, and Thr166 are intact) with fT172A in the presence of ATP. Subsequently, MBP was added for determining the substrate phosphorylation activity of the kinase mixture. As anticipated, WT was able to autophosphorylate, and efficiently trans-autophosphorylate fT172A (**Figures 3B,C**). However, despite the marginal phosphorylation background, fT172A and T172D did not show autophosphorylation and substrate phosphorylation activities, in line with the aforementioned results (**Figure 2**). In addition, these two variants could not trans-autophosphorylate each other (**Figures 3B–D**). Although 5M shares similar overall folding with WT Stk1-KD (Supplementary Figure S3), it barely showed autophosphorylation and substrate phosphorylation activities (**Figures 3B,D**), which may be due to that substitution of Ser/Thr with five alanines hinders the allosteric regulation of Stk1 activation loop during autophosphorylation. However, 5M could still significantly trans-autophosphorylate fT172A, although less efficiently than WT (**Figures 3B,C**). Interaction between 5M and fT172A may trigger the trans-autophosphorylation activity of 5M variant. Interestingly, in comparison with 5M, the mixture of 5M and fT172A did not exhibit increased substrate phosphorylation activity for MBP (**Figures 3B,D**). Moreover, the phosphorylation of MBP in the mixture of WT and fT172A significantly reduced compared to that in the mixture of WT (**Figures 3B,D**), further confirming that phosphorylation of Thr172 is necessary for kinase activity of Stk1. A possible explanation is that the phosphorylated fT172A proteins compete against WT proteins for binding the substrate. Without phosphorylation of Thr172, phosphorylated fT172A is unable to regain activity. Together, these results suggest that autophosphorylation of Thr172 in the GT/S motif is mediated by a *cis* mechanism, while other activation loop serines/threonines are autophosphorylated by a *trans* mechanism.

### Thr172 Is Crucial for Autophosphorylation Activity of Stk1 in *E. coli*

**FIGURE 4:**
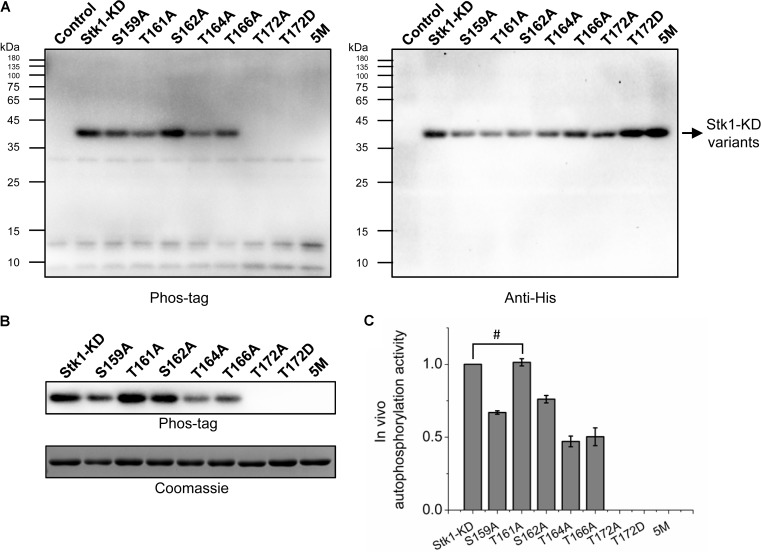
Autophosphorylation of Stk1 activation loop variants *in vivo*. **(A)** Western blotting analysis of *Escherichia coli* cells overexpressing the indicated Stk1-KD variants or pET28a vector alone (Control). Protein phosphorylation was probed using Phos-tag. Expression of Stk1-KD variants was detected using an anti-His antibody. **(B)** Western blotting analysis of purified Stk1-KD variants. Similar amounts of protein kinases were subjected to electrophoresis on a 12% SDS-PAGE gel and stained by Coomassie R250. **(C)** Relative quantification of *in vivo* autophosphorylation level of Stk1-KD variants. Data are represented as the mean ± SEM, *n* = 3 independent experiments. ^#^*P* > 0.05; two-tailed Student’s *t*-test.

The importance of activation loop serines/threonines *in vitro* encouraged us to explore their effects on activity of Stk1 *in vivo*. We utilized *Escherichia coli* as a surrogate *in vivo* context for *S. aureus*. We transformed *E. coli* BL21 (DE3) pLysS cells with pET28a constructs carrying coding sequences of different Stk1-KD variants. *E. coli* BL21 (DE3) pLysS cells transformed with pET28a vector served as a negative control. The expression of Stk1-KD variants were induced by IPTG, and total protein lysates were prepared by sonication. Despite the different expression levels of Stk1-KD variants, T172A, T172D, and 5M variants did not show autophosphorylation activities in the cellular content (**Figure 4A**). To further investigate the impacts of other activation loop serines/theronines on the autophosphorylation activity of Stk1-KD, we directly evaluated the autophosphorylation levels of purified Stk1-KD variants. As expected, T172A, T172D, and 5M variants completely lost autophosphorylation abilities (**Figures 4B,C**). While Ala substitution of Thr161 did not affect the *in vivo* autophsophorylation activity of Stk1-KD, the other kinase variants S159A, S162A, T164A, and T166A showed reduced autophosphorylation abilities in the cellular content (**Figures 4B,C**). Collectively, Thr172 plays an essential role in the autophosphorylation activity of Stk1 *in vivo*.

## Discussion

Signaling systems commonly regulate cellular processes in bacteria. Aside from TCSs, mounting attention is being paid to the Ser/Thr kinases and their cognate phosphatases. Recent works have revealed that modulation of cellular processes by eSTKs/eSTPs is widespread in bacteria including *S. aureus* (Ohlsen and Donat, 2010; Burnside and Rajagopal, 2011; Pereira et al., 2011; Manuse et al., 2016). As a core regulatory module, Stk1/Stp1 are important for *S. aureus* pathogenesis and survival as they are believed to participate in regulating virulence, cell wall structure, and antibiotic resistance (Burnside et al., 2010; Ohlsen and Donat, 2010; Cameron et al., 2012; Sun et al., 2012). Proper function of Stk1/Stp1 pair regulates *S. aureus* virulence, as the reversible phosphorylation events modulated by the pair are essential for controlling bacterial survival in the host (Burnside et al., 2010; Sun et al., 2012). Although there are some strain-to-strain variations in function of Stk1/Stp1, for clinical isolates, it has been established that Stp1 is a positive virulence regulator and a druggable target for *S. aureus* (Burnside et al., 2010; Cameron et al., 2012; Sun et al., 2012; Zheng et al., 2015, 2016; Cai et al., 2017), while Stk1 serves as a negative regulator (Burnside et al., 2010; Tamber et al., 2010). Paradoxically, MRSAs deficient in expression of Stk1 become susceptible to β-lactam antibiotics (Beltramini et al., 2009; Tamber et al., 2010). And the discovery of Stk1 inhibitors further underscores Stk1 as a promising target for combination therapy (Vornhagen et al., 2015; Kant et al., 2017). However, the molecular basis for regulation of its kinase activity remains unknown.

Our current study focuses on investigating the autophosphorylation mechanism of Stk1 activity from *S. aureus*. We initiated the phosphoproteomic study in order to identify the physiological phosphorylation sites in Stk1. Six phosphorylated serines/threonines (Ser159, Thr161, Ser162, Thr164, Thr166, and Thr172) from the activation loop were successfully identified (**Figure 1**), indicative of a fully phosphorylated activation loop within Stk1 in the pathogen. Interestingly, these six phosphorylation sites could be identified after *in vitro* autophosphorylation of Stk1-KD (Supplementary Figure S2), or full-length Stk1 (data not shown), suggesting a similar autophosphorylation manner of Stk1 both *in vitro* and *in vivo*. Without perturbing the overall folding of kinase variants (Supplementary Figure S3), Ala replacement of Stk1 activation loop serines/threonines attenuates autophosphorylation and substrate phosphorylation activities of kinase variants in various degrees (**Figure 2**), suggesting all of activation loop serines/threonines are required for the efficient catalytic activity of Stk1. This also implies that differential phosphorylation of activation loop modulates the kinase activity of Stk1. Interestingly, Ala or Asp substitution of Thr172 in GT/S motif results in non-functional kinases (**Figure 2**), indicating that phosphorylation of Thr172 is essential for activation and kinase activity of Stk1. Indeed, the necessity of phosphorylation of corresponding serine or threonine for kinase activity has also been reported with *M. tuberculosis* Mtb-PknA and *B. anthracis* Ba-Stk1 (Shakir et al., 2010; Ravala et al., 2015), and for eukaryotic kinases (Luciano et al., 2004; Beenstock et al., 2016), implying the conserved function of phosphorylation of GT/S motif.

Subsequent homology modeling of Stk1-KD revealed that Thr172 is the closest residue to the γ-phosphate group of ATP compared to other activation loop serines/threonines. These imply that Stk1 adopts a *cis* autophosphorylation mechanism to transfer the phosphate from ATP to Thr172 to evoke the kinase activity. This speculation could be supported by the amino acid substitution studies for Thr172 (**Figure 2**). Further evidence comes from the trans-autophosphorylation assays. fT172A and T172D did not show autophosphorylation activity, or trans-autophosphorylation activity to each other. However, WT Stk1-KD could autophosphorylate, and efficiently trans-autophosphorylate fT172A and phosphorylate MBP (**Figures 3B,C**). To a less extent, similar results were also observed for 5M where Thr172 is intact. These results suggest that autophosphorylation of Thr172 in the GT/S motif is mediated by a *cis* mechanism, whereas other activation loop serines/threonines are *trans* autophosphorylated. Indeed, similar mechanism was also observed for Thr180 of Mtb-PknA [28].

Ala replacement of serines/threonines in the GT/S motif of homologous Ser/Thr kinases led to inactive variants, which hinders evaluating the effect of phosphorylation of other activation loop residues on kinase activity in the absence of phosphorylation of GT/S motif. For Stk1, the trans-autophosphorylation of fT172A by 5M or WT may provide some clues. 5M could trans-auotophosphorylate fT172A, which did not increase phosphorylation for substrate MBP (**Figures 3B–D**). Unexpectedly, although WT was able to more efficiently trans-auotophosphorylate fT172A, phosphorylated fT172A significantly decreased the phosphorylation level of MBP (**Figures 3B–D**). These results suggest that in the absence of phosphorylated Thr172, phosphorylation of other activation loop residues has no impact on the kinase activity of Stk1. This further supports that phosphorylation of Thr172 is the first step to activate Stk1. The importance of Thr172 was further confirmed by evaluating the autophosphorylation activity of Stk1-KD variants in *E. coli* cells, where T172A or T172D did not show autophosphorylation ability in the cellular content (**Figure 4**), indicating the essentiality of Thr172 for Stk1 activity both *in vitro* and *in vivo*.

**FIGURE 5:**
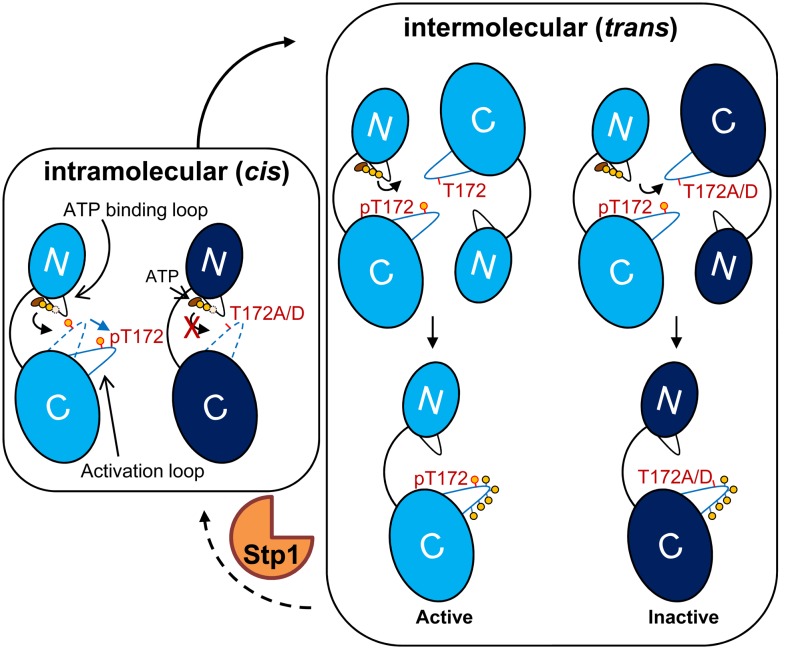
Model for activation of Stk1. The kinase domain of Stk1 (Stk1-KD) contains an N-lobe with an ATP binding loop (black) and a C-lobe with an activation loop (blue). WT Stk1-KD is colored in light blue and T172A variant or T172D variant is colored in dark blue. **(Left)** In the inactive state, the conformation of unphosphorylated activation loop (dotted blue line) mimics a substrate, preventing the binding of substrates. The binding of ATP would trigger the *cis* autophosphorylation of Thr172 and subsequent conformational change of the activation loop (dotted blue line to solid blue line), leading to self-activation of Stk1. **(Right)** Interaction of the activated kinase molecule (pThr172) with another kinase molecule would induce *trans* autophosphorylation of other activation loop serines/threonines for optimal kinase activity. Mutation of Thr172 leads to inactive kinase. Dephosphorylation of Stk1 by Stp1 regenerates the inactive, unphosphorylated kinase. ‘p’ or yellow circles indicate phosphorylated residues.

Based on our current study, we propose an activation model for Stk1 (**Figure 5**). In the inactive state, the unphosphorylated activation loop is folded into the catalytic cleft mimicking a substrate, which prevents the binding of substrates. Under unknown external stimulation, the binding of ATP would trigger the *cis* autophosphorylation of Thr172 to change the conformation of activation loop, leading to self-activation of Stk1. Subsequent interaction with another Stk1 molecule would induce the *trans* autophosphorylation of other activation loop residues for optimal kinase activity. Mutation of Thr172 leads to non-functional kinase, regardless of the phosphorylation status of other activation loop residues. The cognate phosphatase Stp1 dephosphorylates Stk1, regenerating the inactive, unphosphorylated kinase.

In summary, our findings established that the phosphorylation of Thr172 of the GT/S motif in the activation loop is necessary for self-activation and kinase activity of Stk1 from *S. aureus*. Given the conservation of the GT/S motif among the RD family of eSTKs, our findings also provide more insights into the regulation of activity of eSTKs. Thr172 and the stepwise activation of Stk1 may play key roles in virulence and pathogenesis of *S. aureus*, which awaits further studies. Kinases are well-known targets with similar structures, which may hamper the development of specific inhibitors. However, considering the high sequence variability and differential phosphorylation in activation loop of bacterial STKs, this study is also of practical importance for rational design of selective and effective Stk1 inhibitors for combating drug-resistant *S. aureus* including MRSA.

## Author Contributions

WZ, XC, and ZL designed the research. WZ, XC, and SL performed the research. WZ and XC analyzed the data. WZ wrote the manuscript. ZL reviewed and modified the manuscript. All the authors revised and approved the paper.

## Conflict of Interest Statement

The authors declare that the research was conducted in the absence of any commercial or financial relationships that could be construed as a potential conflict of interest.

## Footnotes

1 https://www.ebi.ac.uk/pride/archive/
