# Supplementary material for: Autophosphorylation Mechanism of the Ser/Thr Kinase Stk1 From Staphylococcus aureus
Source: Front Microbiol. 2018 Apr 20;9:758. doi: 10.3389/fmicb.2018.00758 (PMC5920020; doi:10.3389/fmicb.2018.00758)
Supplement: Supplementary file 1 [file Presentation_1.pdf]

Supplementary Material

Autophosphorylation mechanism of the Ser/Thr kinase Stk1 from *Staphylococcus aureus*

Weihao Zheng[1], Xiaodan Cai[1], Shuiming Li[2], and Zigang Li[1*]

[1] School of Chemical Biology and Biotechnology, Peking University Shenzhen Graduate School, China
[2] College of Life Sciences and Oceanography, Shenzhen University, China

*** Correspondence:** Zigang Li, lizg@pkusz.edu.cn

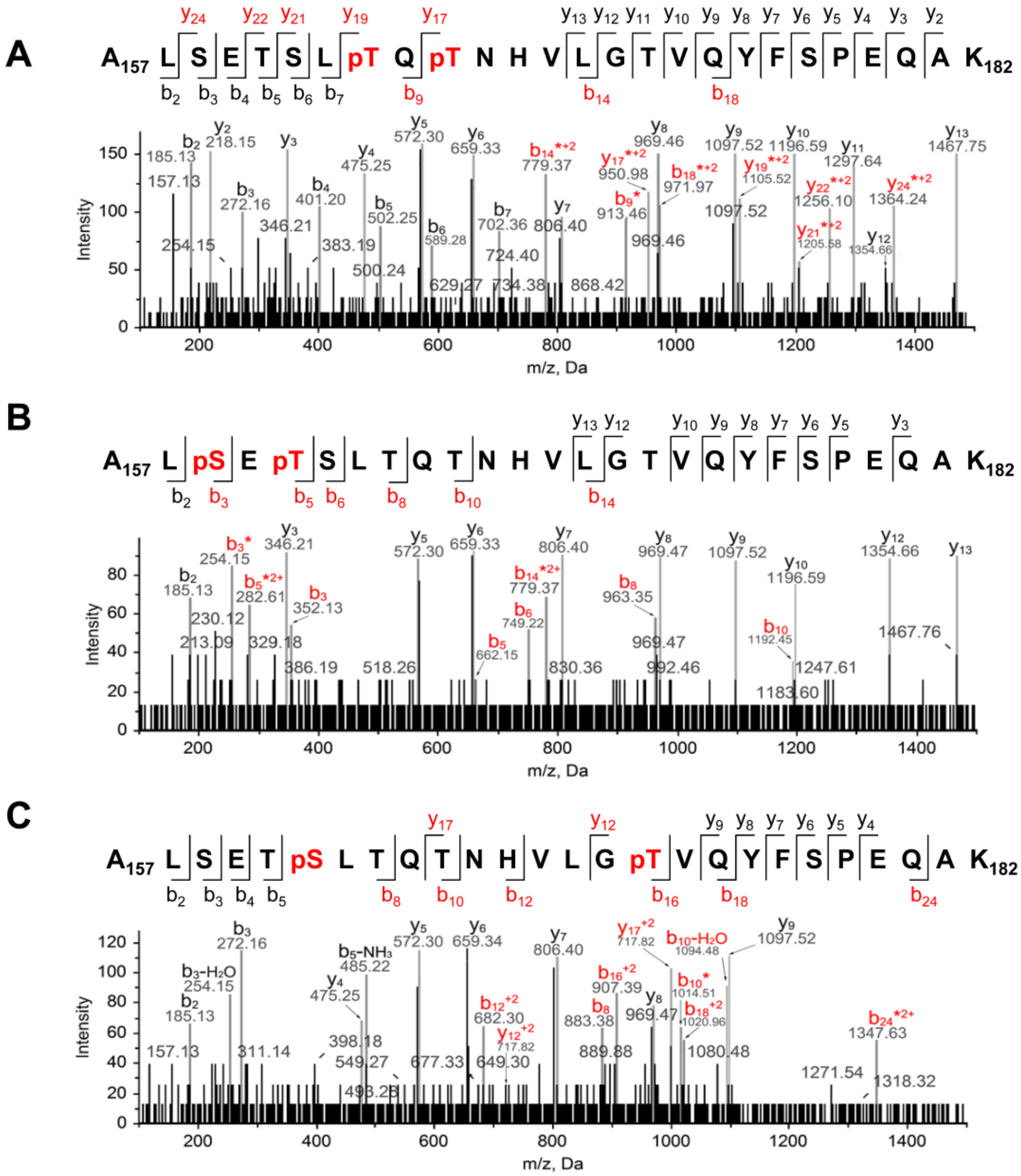

FIGURE S1 Identification of *in vivo* phosphorylated residues within the activation loop of Stk1. Phosphoproteomic study showed that Stk1 were phosphorylated at Thr164/Thr166 **(A)**, Ser159/Thr161 **(B)** and Ser162/Thr172 **(C)**. Different ‘b’ and ‘y’ fragment ions are indicated. Fragment ions with the loss of phosphoric acid (−98 Da) are marked with an asterisk (*).

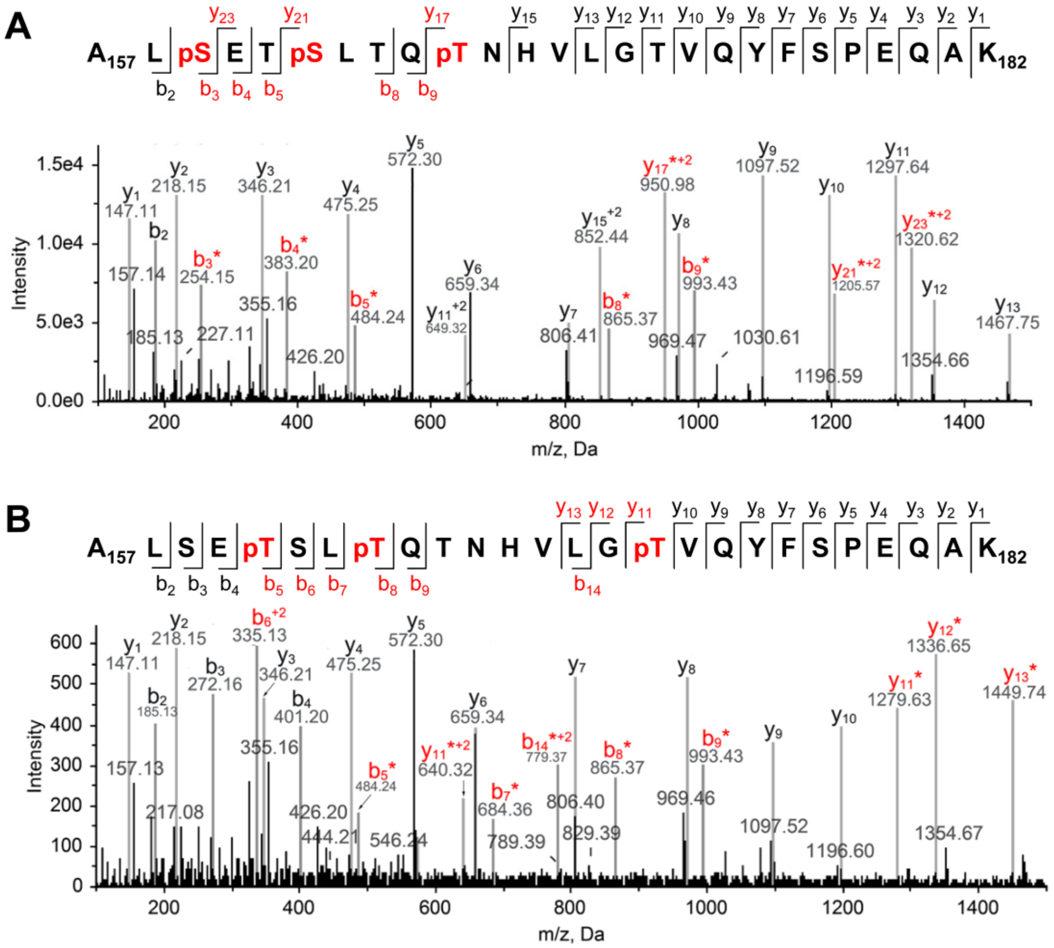

FIGURE S2 Identification of *in vitro* phosphorylated residues within the activation loop of Stk1. MS/MS spectra showed that Stk1 were autophosphorylated at Ser159/Ser162/Thr166 **(A)** and Thr161/Thr164/Thr172 **(B)** in the activation loop. Different 'b' and 'y' fragment ions are indicated. Fragment ions with the loss of phosphoric acid (−98 Da) are marked with an asterisk (*).

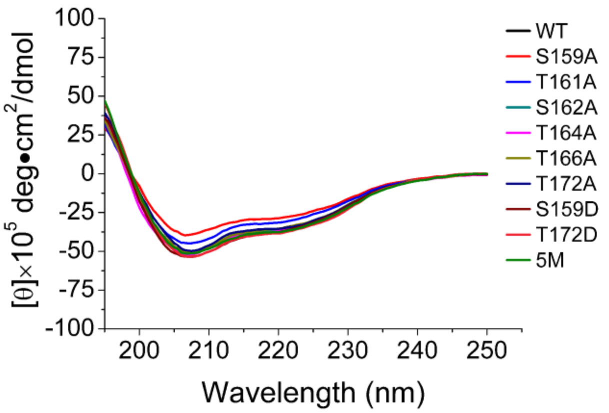

FIGURE S3 The far-UV (195–250 nm) CD spectra of WT Stk1-KD and its cognate variants. Ala substitution of Stk1 activation loop serines/threonines caused minor changes in secondary structure content of the kinase variants.

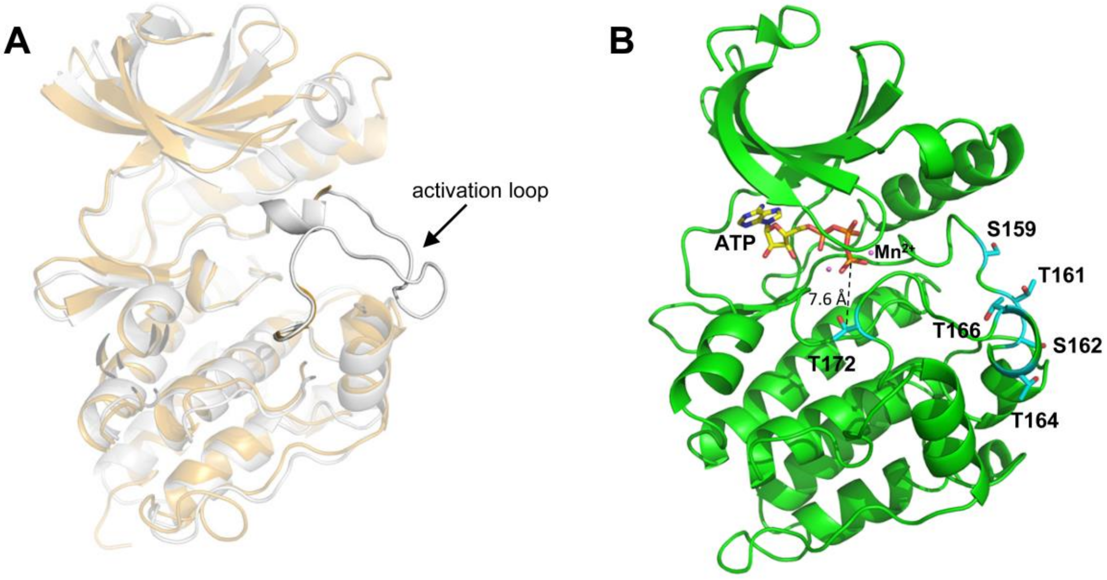

FIGURE S4 (A) Overlap of the Stk1-KD structure (colored gold; PDB code: 4EQM) and Stk1-KD model (colored white; built with Swiss-model). **(B)** The autophosphorylated residues in the activation loop of Stk1 are labeled in cyan. Thr172 is the nearest residue to the γ-phosphate group of ATP (7.6 Å) compared to other activation loop serines/threonines (Ser159: 14.5 Å, Thr161: 17.4 Å, Ser162: 19.7 Å, Thr164: 21.5 Å, Thr166: 15.8 Å). Modeling of ATP and Mn^{2+} from cAMP-dependent protein kinase (PDB code: 1ATP) into the active site of Stk1-KD was conducted in MOE (Molecular operating environment) software.

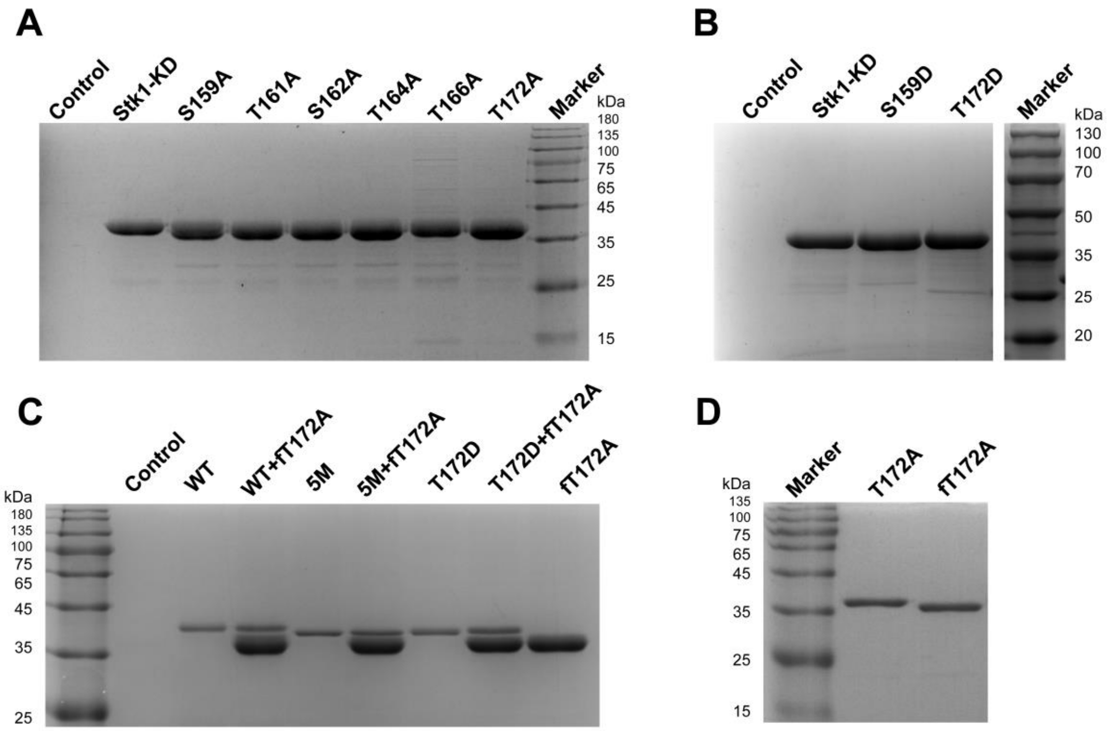

FIGURE S5 Original images for the SDS-PAGE gels stained with Coomassie R250 (Coomassie) in Figure 2A **(A)**, Figure 2D **(B)** and Figure 3B **(C)**. **(D)** SDS-PAGE analysis of purified His-tag-free T172A (fT172A).

Table S1 Primers used for cloning and mutagenesis in this study

Primers	Sequence (5'-3')	Application
Stk1KD-NdeI-F	GTGTACACATATGATAGGTAAAATAATAAATGAACG	Protein cloning
Stk1KD-XhoI-R	GTAGGCACTCGAGTTATTCTTTTTTCAAAGGTACCGC	Protein cloning
S159A-F	GAATTGCTAAAGCTTTAGCTGAGACGTCTTTAACTCAGACTAA	Mutagenesis
S159A-R	GAGTTAAAGACGTCTCAGCTAAAGCTTTAGCAATTCCAAAATC	Mutagenesis
T161A-F	GCTAAAGCTTTAAGTGAGGCGTCTTTAACTCAGACTAATCATGT	Mutagenesis
T161A-R	TTAGTCTGAGTTAAAGACGCCTCACTTAAAGCTTTAGCAATTCC	Mutagenesis
S162A-F	GCTAAAGCTTTAAGTGAGACGGCTTTAACTCAGACTAATCATGTG TTA	Mutagenesis
S162A-R	GATTAGTCTGAGTTAAAGCCGTCTCACTTAAAGCTTTAGCAATTC C	Mutagenesis
T164A-F	AAGTGAGACGTCTTTAGCTCAGACTAATCATGTGTTAGGTAC	Mutagenesis
T164A-R	GATTAGTCTGAGCTAAAGACGTCTCACTTAAAGCTTTAGCA	Mutagenesis
T166A-F	GTGAGACGTCTTTAACTCAGGCTAATCATGTGTTAGGTACTGTGC AGT	Mutagenesis
T166A-R	GTACCTAACACATGATTAGCCTGAGTTAAAGACGTCTCACTTAAA GC	Mutagenesis
T172A-F	CAGACTAATCATGTGTTAGGTGCTGTGCAGTACTTTTCGCCAGAA CAAG	Mutagenesis
T172A-R	GGCGAAAAGTACTGCACAGCACCTAACACATGATTAGTCTGAGT TAAAG	Mutagenesis
S159D-F	ATTGCTAAAGCTTTAGATGAGACGTCTTTAACTCAGACT	Mutagenesis
S159D-R	AAGACGTCTCATCTAAAGCTTTAGCAATTCCAAAATCA	Mutagenesis
T172D-F	TAATCATGTGTTAGGTGATGTGCAGTACTTTTCGCCAGA	Mutagenesis
T172D-R	AGTACTGCACATCACCTAACACATGATTAGTCTGAGTTA	Mutagenesis
5M -F	GCTTTAGCTGAGGCGGCTTTAGCTCAGGCTAATCATGTGTTAGGT AC	Mutagenesis
5M -R	CTAACACATGATTAGCCTGAGCTAAAGCCGCCTCAGCTAAAGCT TTAGC	Mutagenesis

Table S2 Identification of putative phosphopeptides and phosphorylated proteins

Genes	Proteins	Phosphopeptides	Phosphorylation sites
map	MHC class II analog protein	AGIYTADLINTSEIK	T134
map	MHC class II analog protein	KDKANYQVPYTITVNGTSQNILSNLTFNK	T167
tufA	translation elongation factor Tu (EF-Tu)	NGDSVAQSYDMIDNAPEEK	S42*
gapA	glyceraldehyde 3-phosphate dehydrogenase 1	AAAENIIPNSTGAAK	S210
NWMN_2086	alkaline shock protein 23	QAYDNQTGVNEK	Y12
NWMN_2086	alkaline shock protein 23	GGLTDTFTNAFSSGNNVTQGVSVEVGEK	T73
fus	translation elongation factor G (EF-G)	YLGDEEISVSELK	S238
fus	translation elongation factor G (EF-G)	VMTDPYVGK	K324
eno	enolase	SGETEDTTIADIAVATNAGQIK	S373
NWMN_1831	ferritin	IDFSSILETFK	S81
NWMN_0783	CsbD-like superfamily protein	ADESKFEQAK	S5
NWMN_0783	CsbD-like superfamily protein	ETVGNVTDNK	T22
NWMN_0783	CsbD-like superfamily protein	ETVGNVTDNK	T17
pykA	pyruvate kinase	ALGLITEENGITSPSAIVGLEK	T537
pykA	pyruvate kinase	ALGLITEENGITSPSAIVGLEK	T531

pykA	pyruvate kinase	ALGLITEENGITSPSAIVGLEK	T537
pykA	pyruvate kinase	ALGLITEENGITSPSAIVGLEK	T537
pykA	pyruvate kinase	ALGLITEENGITSPSAIVGLEK	S538
pykA	pyruvate kinase	ALGLITEENGITSPSAIVGLEK	T531
pykA	pyruvate kinase	ALGLITEENGITSPSAIVGLEK	T537
pykA	pyruvate kinase	ALGLITEENGITSPSAIVGLEK	T537
pykA	pyruvate kinase	ALGLITEENGITSPSAIVGLEK	T537
pykA	pyruvate kinase	ALGLITEENGITSPSAIVGL	S540
pykA	pyruvate kinase	GITSPSAIVGLEK	T537
rplY	50S ribosomal protein L25	NGVIELGVGSK	K68
pgm	2,3-bisphosphoglycerate-independent phosphoglycerate mutase	YPTTQIEASGLDVGLPEGQMGNS EVGHMNIGAGR	S62
pgm	2,3-bisphosphoglycerate-independent phosphoglycerate mutase	YPTTQIEASGLDVGLPEGQMGNS EVGHMNIGAGR	S48
pgm	2,3-bisphosphoglycerate-independent phosphoglycerate mutase	YPTTQIEASGLDVGLPEGQMGNS EVGHMNIGAGR	S62
pgm	2,3-bisphosphoglycerate-independent phosphoglycerate mutase	YPTTQIEASGLDVGLPEGQMGNS EVGHMNIGAGR	S62
pgm	2,3-bisphosphoglycerate-independent phosphoglycerate mutase	YPTTQIEASGLDVGLPEGQMGNS EVGHMN	S62
pgm	2,3-bisphosphoglycerate-independent phosphoglycerate mutase	YPTTQIEASGLDVGLPEGQMGNS EVGHMNIGAGR	S62
pgm	2,3-bisphosphoglycerate-independent phosphoglycerate mutase	YPTTQIEASGLDVGLPEGQMGNS EVGHMNIGAGR	S62
pgm	2,3-bisphosphoglycerate-independent phosphoglycerate mutase	YPTTQIEASGLDVGLPEGQMGNS EVGHMNIGAGR	S62

pgm	2,3-bisphosphoglycerate-independent phosphoglycerate mutase	YPTTQIEASGLDVGLPEGQMGNS EVGHMNIGAGR	S62
pgm	2,3-bisphosphoglycerate-independent phosphoglycerate mutase	YPTTQIEASGLDVGLPEGQMGNS EVGH	S62
NWMN_152 6	hypothetical protein	SFMDK	S2
NWMN_152 6	hypothetical protein	INEYTGSNNEEK	Y32
NWMN_152 6	hypothetical protein	INEYTGSNNEEK	S35
NWMN_250 3	fructose-bisphosphate aldolase class-I	GFIAALDQSGGSTPK	S22
NWMN_250 3	fructose-bisphosphate aldolase class-I	VVVLSGGYSR	S238
fbaA	fructose-bisphosphate aldolase	INVNTENQIASAK	T234
gudB	NAD-specific glutamate dehydrogenase	RDSFGTVTNLFEETISNK	S278
gudB	NAD-specific glutamate dehydrogenase	RDSFGTVTNLFEETISNK	T281
gudB	NAD-specific glutamate dehydrogenase	RDSFGTVTNLFEETISNK	T283
gudB	NAD-specific glutamate dehydrogenase	RDSFGTVTNLFEETISNK	S278
gudB	NAD-specific glutamate dehydrogenase	RDSFGTVTNLFEETISNK	T281
gudB	NAD-specific glutamate dehydrogenase	RDSFGTVTNLFEETISNK	T283
gudB	NAD-specific glutamate dehydrogenase	RDSFGTVTNLFEETISNK	S278
gudB	NAD-specific glutamate dehydrogenase	RDSFGTVTNLFEETISNK	T283
gudB	NAD-specific glutamate dehydrogenase	RDSFGTVTNL	S278
gudB	NAD-specific glutamate dehydrogenase	DSFGTVTNLFEETISNK	S278
gudB	NAD-specific glutamate dehydrogenase	DSFGTVTNLFEETISNK	T281

gudB	NAD-specific glutamate dehydrogenase	DSFGTVTNLFEETISNK	S278
gudB	NAD-specific glutamate dehydrogenase	DSFGTVTNLFEETISNK	S278
gudB	NAD-specific glutamate dehydrogenase	DSFGTVTNLFEETISNK	T283
NWMN_0513	molecular chaperone Hsp31 and glyoxalase 3	SQDVNELSK	S2
NWMN_0513	molecular chaperone Hsp31 and glyoxalase 3	VNELSK	S9
glmM	phosphoglucosamine-mutase	DMGAELGVMISASHNPVADNGIK	S102
rsbV	anti-sigma B factor antagonist	DIYVNLENVSYMDSTGLGLFVGTLK	S57
rsbV	anti-sigma B factor antagonist	DSTGLGLFVGTLK	S57
NWMN_1818	conserved hypothetical protein	LSPSEVTAIADALGQLR	S51
pgi	glucose-6-phosphate isomerase	SGTTTEPAVAFR	T143
guaA	glutamine-hydrolyzing GMP synthase	EIWQYFTVLPN	T437
glyA	serine hydroxymethyltransferase	YAEGYPGR	Y51
rpmC	50S ribosomal protein L29	DLTTSEIEEQIK	S16
upp	uracil phosphoribosyltransferase	LSYIRDVNTGTKEFR	T24
NWMN_2282	general stress protein	SNSQAIQAIENVLATSK	S4
NWMN_1518	conserved hypothetical protein	MENFDKTMK	T7
stk1	serine/threonine-protein kinase	ALSETSLTQTNHVLGTVQYFSPEQAK	T164; T166
stk1	serine/threonine-protein kinase	ALSETSLTQTNHVLGTVQYFSPEQAK	S159; T161

stk1	serine/threonine-protein kinase	ALSETSLTQTNHVLGTVQYFSPE QAK	S162; T172
stk1	serine/threonine-protein kinase	ALSETSLTQTNHVLGTVQYFSPE QAK	T166; T172
stk1	serine/threonine-protein kinase	ALSETSLTQTNHVLGTVQYFSPE QAK	T166
hprK	HPr kinase/phosphatase	LNIMGINTAEEFSER	S294
capB	capsular polysaccharide synthesis enzyme CapB	SSSYYHYYGDE	Y227
capB	capsular polysaccharide synthesis enzyme CapB	SSSYYHYYGDE	Y227; Y228

*Phosphorylation sites (colored in red) of present study have been confirmed by other phosphoproteomic studies.
